# 454 sequencing of pooled BAC clones on chromosome 3H of barley

**DOI:** 10.1186/1471-2164-12-246

**Published:** 2011-05-19

**Authors:** Kazuhiro Sato, Yuka Motoi, Nami Yamaji, Hideya Yoshida

**Affiliations:** 1 Institute of Plant Science and Resources, Okayama University, Kurashiki, 710-0046, Japan

## Abstract

**Background:**

Genome sequencing of barley has been delayed due to its large genome size (ca. 5,000Mbp). Among the fast sequencing systems, 454 liquid phase pyrosequencing provides the longest reads and is the most promising method for BAC clones. Here we report the results of pooled sequencing of BAC clones selected with ESTs genetically mapped to chromosome 3H.

**Results:**

We sequenced pooled barley BAC clones using a 454 parallel genome sequencer. A PCR screening system based on primer sets derived from genetically mapped ESTs on chromosome 3H was used for clone selection in a BAC library developed from cultivar "Haruna Nijo". The DNA samples of 10 or 20 BAC clones were pooled and used for shotgun library development. The homology between contig sequences generated in each pooled library and mapped EST sequences was studied. The number of contigs assigned on chromosome 3H was 372. Their lengths ranged from 1,230 bp to 58,322 bp with an average 14,891 bp. Of these contigs, 240 showed homology and colinearity with the genome sequence of rice chromosome 1. A contig annotation browser supplemented with query search by unique sequence or genetic map position was developed. The identified contigs can be annotated with barley cDNAs and reference sequences on the browser. Homology analysis of these contigs with rice genes indicated that 1,239 rice genes can be assigned to barley contigs by the simple comparison of sequence lengths in both species. Of these genes, 492 are assigned to rice chromosome 1.

**Conclusions:**

We demonstrate the efficiency of sequencing gene rich regions from barley chromosome 3H, with special reference to syntenic relationships with rice chromosome 1.

## Background

Barley (*Hordeum vulgare *L.) is a model genome system for the Triticeae, which includes wheat and rye, since it is a self-fertile diploid (2n = 14) that shares homoeologous chromosomes with other members of the Triticeae [1]. However, genome sequencing of barley has been delayed due to its large genome size (ca. 5,000Mbp) [2]. An alternative genomics resource - a large number of expressed sequence tags (ESTs) - has been developed for barley. Quality controlled EST information is available from the HarvEST:Barley database [3].

Genome wide mapping of these ESTs provides an important framework of genome structure that can be used to approach whole genome sequencing, as demonstrated in rice [4,5]. We have developed a high resolution EST linkage map of barley using progeny of a single cross and 2,890 PCR-based markers [6]. The ESTs were derived from non-redundant 3' sequences, generating a comprehensive distribution of genes on the barley linkage map. This high density EST map provides a foundation for map-based genome analysis by providing a basis for selecting BAC (bacterial artificial chromosome) clones for sequencing [7]. The mapped barley ESTs also provide access to other genomes, such as rice (*Oryza sativa *L.) via homology. For example, an integrated barley transcript map identified micro-colinearity between rice genome sequence and barley ESTs [8,9]. Although the chromosome numbers of barley and rice are different, complete chromosomal colinearity has been reported between barley chromosome 3H and rice chromosome 1 [10,11]. This finding is of particular interest in terms of genome evolution in grasses. It also makes the homoeologous group 3 chromosomes of the Triticeae a logical target for chromosome oriented genome sequencing, using rice as a reference genome [12].

Several BAC libraries have been developed in barley. The first BAC library was developed for the American malting barley cultivar "Morex" [13]. Morex traces to barley germplasm of Manchurian origin and was used as a parent of mapping populations used for extensive linkage and QTL mapping [14]. Another high quality BAC library was developed using the Japanese malting barley cultivar "Haruna Nijo" [15]. Haruna Nijo traces to barley germplasm of European origin and therefore may have a haplotype for brewing-related genes similar to European malting barleys. Since these two representative barley cultivars have different origins, the BAC libraries developed from them should contain the sequence variation that leads to phenotypic variation for quality and agronomic characters.

Our goal is to contribute to this deeper understanding of allelic variation in barley by developing, characterizing, and providing the full complement of genomics tools (ESTs, transcript map, sequenced full-length (FL) cDNAs and BAC library) developed for Haruna Nijo at Okayama University. Morex will be used as the target haplotype by the international barley sequencing consortium [16,17]. The simultaneous sequencing of two haplotypes will be much more useful than single haplotype analysis, as it will reveal the basis of structural and functional allelic diversity within the species.

Recent advances in high-throughput sequencing are based on bead capture and parallel sequencing reads. However, read lengths are still short compared to traditional Sanger sequencing. Among the fast reading systems, liquid phase pyrosequencing [18] provides the longest reads and is the most promising method for BAC clones [19]. Here we report the results of pooled sequencing of BAC clones selected with ESTs genetically mapped to chromosome 3H.

## Results and Discussion

### BAC clone sequencing efficiency

**Figure 1:**
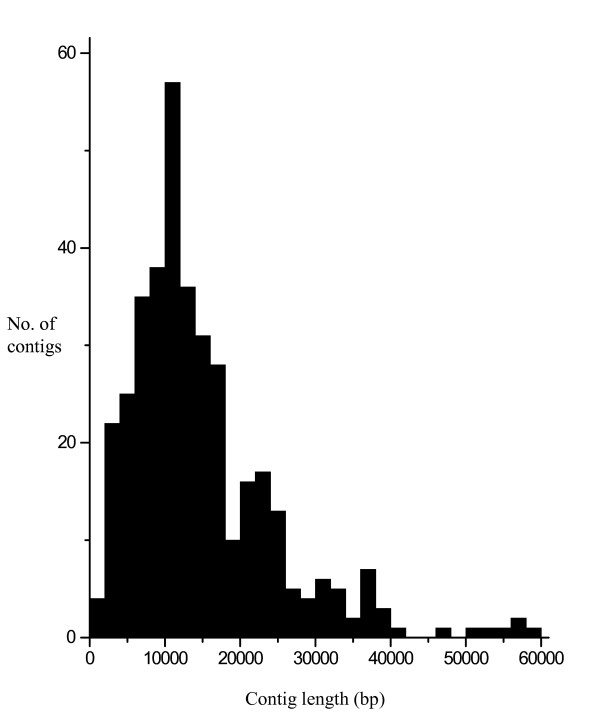
**Distribution of contig lengths identified by mapped EST sequences on barley chromosome 3H**. Contig lengths ranged from 1,230bp to 58,322bp, with an average 14,891bp.

444 EST markers were used to select 400 BAC clones from a Haruna Nijo pooled genomic library that were then chosen for sequencing. The insert size of 400 BAC clones ranged from 20 kb to 284 kb, with an average of 118 kb. The minimum redundancy of sequence reads was 30 x, making for a standard read of 4 Mb per BAC clone (see Additional file 1). After trimming BAC vector (pBAC-Lac [15]) and *E. coli *K12 genome sequences, there were 7,512 contigs greater than 500 bp, meaning that on average 19 contigs were obtained from each clone. Contig lengths ranged from 500 bp to 58,322 bp, with an average 3,678 bp. The tentative number of barley unigenes is 32,690, based on CAP3 assembly [20] of 3' end ESTs and FLcDNAs [21]. The number of contigs with significant blastn [22] homology (E < 1*E*-30) with these tentative unigenes was 3,854. In some cases, only short sequence segments in the contigs show homology to the unigenes; thus the number of gene-bearing contigs may actually be lower. The average number of unigenes per contig was 5.1. There was no correlation between contig length and number of unigenes per contig (data not shown). Of the 444 EST sequences used for the BAC clone selection, 393 ESTs allowed the selection of 400 BAC clones, as seven ESTs were used to select first and second BAC clones due to an insufficient insert size of first BAC clone in the initial pooled library. Of the 393 ESTs, 372 were identified on the contigs (see Additional file 2). The contigs that show homology ranged from 1,230 bp to 58,322 bp with an average of 14,891 bp (Figure 1, see also Additional file 2).

There are probably 30,000-40,000 genes in barley, based on the total number of expressed rice genes (31,439; Rice Annotation Project (RAP) Database [23]) and the 32,690 barley unigene estimate cited above. The number of genes on chromosome 3H, as calculated by simple division, would be 6,000-7,000. This estimate is lent credence by the reported number of genes on wheat chromosome 3B [12]. Chromosome 3H may be ~ 700 Mbp in length, based on simple division of total genome size (ca. 5,000 Mbp [2]) by the chromosome number (n = 7). Based on these rough calculations one would expect ~ 10 genes/Mb. The total contig sequence length of the present analysis is ~ 28 Mb. As indicated later, number of rice ORFs identified on the total contigs was 1,239 (see Additional file 3). This indicates 44 gene candidates are estimated per Mb. Even factoring in overestimation of gene number due to incorrect homology, the number of gene candidates identified was far more than the predicted number. This may indicate a highly efficient rate of gene discovery.

**Figure 2:**
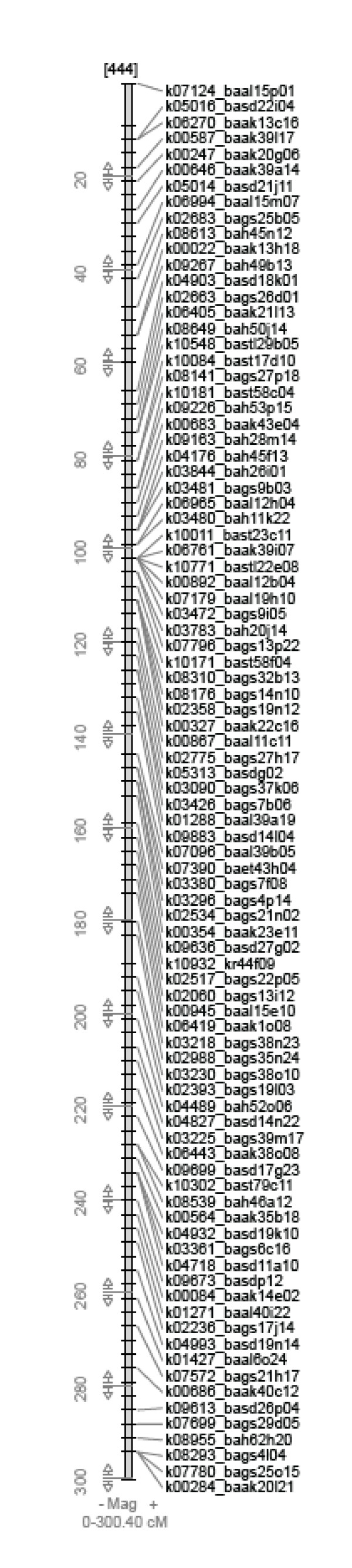
**A platform of sequence query from skeletal presentation of cMAP on barley chromosome 3H **[6]. Blastn hit contig sequences are listed to link with database searches in Gbrowse annotation system (available online [31]).

On the other hand, the total read length of 28 Mbp represents ~ 4% of chromosome 3H and is much less than the total length of the BAC sequences (48.8 Mbp based on 400 clones with an average insert size of 118 kb). The shorter total read length may indicate sequence overlaps between the BAC clones which were selected by genetically mapped ESTs (Figure 2). A massive translocation line study [24] estimated that 47.3% of the genetic markers mapped to recombination hot spots representing only 4.9% of the barley genome. Thus, BAC selection by genetically mapped markers may lead to an inevitably high level of sequence duplication. We also expected sequence duplication in the current analysis since mapped ESTs appear in several dense clusters on the same genetic positions in Figure 2 and seven of these ESTs were used to select multiple BAC clones. Some efficiencies might be achieved by using physically mapped markers [24].

### Homology with rice genes and the rice genome

**Figure 3:**
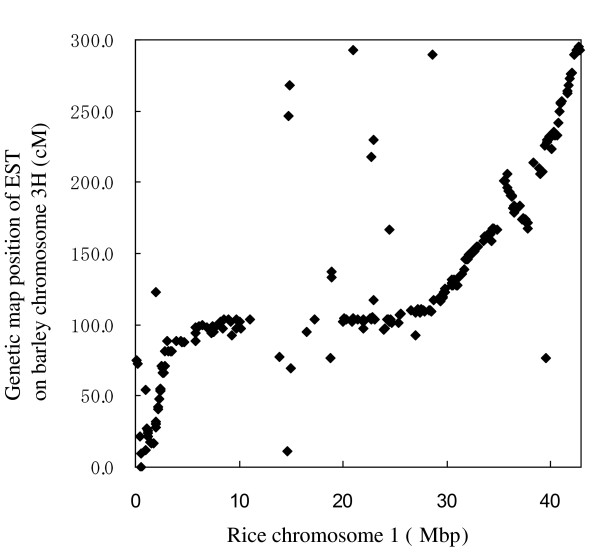
**Visualization of colinearity between contigs homologous to mapped barley EST on chromosome 3H **[6]**and rice chromosome 1 genome sequence (Rap2 pseudomolecule **[23]**)**.

The RAP2 rice pseudomolecule [23] was used as a basis for homology search with the 372 contigs identified by ESTs mapped to barley chromosome 3H (see Additional file 2). Of these contigs, 240 showed significant blast scores (E < 1*E*-20) with sequences on rice chromosome 1. A comparison of genetic map positions with the rice genome is shown in Figure 3. There is clear colinearity between the two species, except for the centromeric region. There is some evidence for an inversion on the long arm; additional detailed sequence analysis will be required. This high level of colinearity indicates that sequences from rice chromosome 1 can be used very efficiently for identification of sequences on barley chromosome 3H.

All 31,439 RAP2 rice gene nucleotide sequences [23] were used to determine homology with all barley sequence contigs greater than 500 bp. Six percent (1,790) of the genes in rice showed homology (E < 1*E*-20) with these contigs (see Additional file 4). Barley regions showing homology with rice genes (rap2 nucleotide sequences in each locus [23]) were also analyzed. The existence of full rice gene sequences on barley contig is confirmed by the start and end positions of rice gene on the respective contig sequence. This evaluation revealed 1,239 rice homologous genes on barley contigs (see Additional file 3). Of these rice genes, 492 are assigned to rice chromosome 1, indicating that our preliminary sequencing of barley chromosome 3H BAC clones have homology with 12% of the genes on rice chromosome 1.

One of the aims of this BAC clone sequencing project was the rapid identification of genes in contig sequences. Since the number of barley FLcDNAs available for this purpose (5,006) is limited compared to the total number of barley genes, the efficiency of barley gene identification was estimated based on homologous rice genes. The large number of rice gene homologous sequences (1,239) (see Additional file 3) identified in barley contigs is a parallel line of evidence for our high rate of barley gene discovery. Conversely, the high number of barley genes assigned to chromosome 1 of rice (492) indicates that the focused sequencing of the barley genome using rice genome resources is an effective strategy. Specifically, an efficient barley genome sequencing strategy can be based on sequencing EST-positive BAC pools using a filtration system followed by the confirmation with homologous rice genes.

As a more closely related reference genome to barley, a set of coding sequences of *Brachypodium distachyon *[25] were searched for homology with the contig sequences (see Additional file 5). The number of sequences showing homology to the barley contigs was 2,050 (E < 1*E*-20) and higher than that of rice (1,790). When a detailed annotation of the *Brachypodium *genome is available, it will be a valuable resource for barley genetics since *Brachypodium *is more closely related to barley than rice.

### 454 sequencing capacity

The sequencing capacity of 454 is far greater than a high-throughput Sanger system. However, the combination of shorter read length and BAC clone pooling used in this study could cause problems for sequence assembly. If the read length is 100 bp by GS20, an assembly error may occur when sequence repeats more than 100 bp are present. To avoid miss-assembly, all pool libraries contained FLX reads (average read length 250bp). Survey sequencing [19] demonstrated the robustness of sequence assembly using the 454 GS20 (100 bp average read length) by comparing sequences with those obtained with a Sanger sequencing system. Pooled BAC sequencing technology by 454 was also used to assess the feasibility of sequencing BAC pools of Atlantic salmon [26]), melon [27]) and rice [28]. There may be a certain level of assembly error in the sequences we report from this study that could be corrected in the future by repeating reads in the different haplotypes, or by generating scaffolds with a paired-end sequencing system [26].

Another issue with our sequencing strategy is identifying each BAC clone in the pooled shotgun library. A technique to identify each clone by short tag sequences is becoming available (e.g. barcoding reads from each BAC clone) [29]. However, shotgun library development for each clone is costly and limits sequencing capacity. The pooled BAC sequencing strategy used in this study is a pioneering step for barley and the Triticeae and it may be the most efficient strategy by the current technical standards.

### Development and function of a genome browsing system

**Figure 4:**
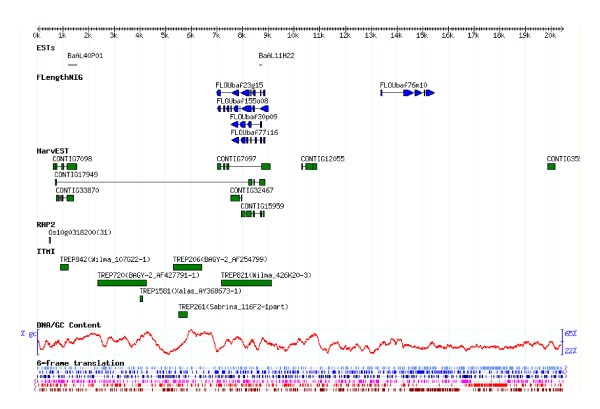
**An example screenshot of GBrowse mapping of barley cDNAs on a sequenced contig**. Several barley full length cDNAs mapped on 7-9kb region, indicating possible alternatively spliced transcripts from a single gene. Gbrowse access is available online [31].

The 8,583 contigs with sequences longer than 30 bp were aligned with reference sequences installed on the Gbrowse system [30] (online access available [31]). The browser also has search functions using sequence queries and mapped ESTs (Figure 2, see also Additional file 2) on a cMAP browser [32]. The blastn analysis using FLcDNAs gave 1,474 contigs greater than 500 bp with significant blastn scores (E < 1*E*-30). Of these, 453 contigs showed homology to multiple FLcDNA clone sequences, providing evidence for possible alternative splicing of some genes (see Additional file 6). An example of Gbrowse alignment by mapping homologous regions from multiple FLcDNAs on a contig is shown in Figure 4.

Browser functions will be expanded as more genome sequences are deposited. The flexible connections that are provided to connect GeneChip expression data, genetic map data, and cDNA data will promote rapid isolation of barley genes and analysis of their functions. As demonstrated by the cMAP query function in the current browser (Figure 2), the combination of genetic map and partial genome sequence is a framework for genome sequencing of gene rich regions in barley.

Haplotype information for breeding materials and genetic stocks is important for plant breeders [33]. Even if only sparse genome information is available, comparisons of sequences in diverse germplasm may provide important information for crop improvement. Thus, one of the ultimate functions of a barley genome browser may be to reveal critical sequence polymorphisms in germplasm. The release of partial genome sequences of Morex BAC clones (Stein et al. personal communication) will, in the near future, demonstrate this genome browser utility.

## Conclusions

Our results for Haruna Nijo chromosome 3H are the first comprehensive genome sequence information for barley. Since there are additional markers on chromosome 3H mapped in other germplasm [34] (see also HarvEST database [3]), more 3H BAC clones could be sequenced. The haplotype sequence of Haruna Nijo will likely be quite different from that of Morex, which will be used by the international barley genome sequencing consortium [16]. Therefore, the availability of multiple haplotypes will result in complementary information on genome structure that will provide a basis for efficient polymorphism detection.

In spite of recent innovations in genome sequencing, a BAC-by-BAC sequencing strategy for barley is not efficient. A possible approach will be to combine sequencing of EST bearing-BACs and shotgun whole genome, or chromosome specific, libraries [35]. The sequencing and annotation system used in this study will also be applicable, except for the assembly of highly redundant reads on a whole genome basis.

## Methods

### BAC clone screening by mapped EST makers on chromosome 3H

A PCR screening system was developed for the plate-pooled and super-pooled DNA of the Haruna Nijo BAC library [15]. Original library plates were copied and cultured on 384-well plates. The samples were transferred to a set of 384 PCR screening plates with linked 24 rows or 16 columns (Assist Co. Ltd. No. A.384SC30). A total of 40 pooled row or column *E. coli *samples per 384-well plate were used to isolate plasmid DNA samples by an automated DNA isolation system (PI-200, Kurabo Industries Ltd.). The procedures were repeated to isolate all the DNA samples in 768 384-well plates.

Primers used for transcript map development [6] were then used to identify BAC clones. Each target BAC clone was screened by PCR on the super-pooled and plate-pooled DNA to identify the plate in which the target BAC clone was present [15]. Then the clone address on the plate was identified by PCR using row/column pooled DNA samples. After the colony PCR of each clone, the EST bearing-BAC clone was confirmed. A total of 444 EST markers were used for BAC clone screening and 400 BACs were identified (see Additional file 7 for EST sequences). Seven ESTs were used to select first and second BAC clones due to an insufficient insert size of first BAC clone in the initial pooled library. The other 51 markers failed to identify BAC clones due to extraordinary number of products or to non-amplification by PCR.

### Library development and sequencing

Individual clones were cultured on LB medium and a small amount of DNA was isolated using an automated plasmid isolation system (PI-200, Kurabo). Insert size was estimated by pulse field gel electrophoresis (CHEF DR-III, Bio-rad Lab. Inc.). Each clone was cultured in a 100 ml flask to harvest plasmids. *E. coli *samples of 10 or 20 clones were mixed and used for isolation and purification of plasmids using the Large-Construct Kit (QIAGEN). 3-5 μg of the pooled plasmid DNA was used for shotgun library development with a library preparation kit (Roche Applied Science) according to the manufacturer's protocol. Information of pooled barley BAC clone libraries and resulting sequences are presented at Additional file 1.

Each library was used for the emulsion PCR amplification. The PCR-amplified fragments on beads were washed and the bead number was counted using a Coulter Counter Z1 single threshold instrument (Beckman Coulter Co.). The appropriate number of beads was applied on a pico titer plate according to the manufacturer's protocol. More than 30 x redundancy was sequenced for each library using a Roche 454 genome sequencer GS20 (average read length 100bp) or FLX (average read length 250bp). The pyrosequencing reaction data were base-called using the software installed on the analysis server of sequencer. Sequences and quality scores were assembled by the Newbler assembler provided by the manufacturer and installed on a 32-bit Linux server. Contig sequences were trimmed for BAC vector sequence and *E. coli *K12 genome sequence. Assembled sequences more than 500bp were used for further analyses.

A small amount of DNA from each clone was used for plasmid amplification using TempliPhi Large Construct Kit (Invitrogen Japan K.K.). BAC end sequences were produced by cycle sequencing using 3130xl genetic analyzer (Applied Biosystems Co.).

### Analysis of contig sequences and development of genome browser

Contig sequences generated in each pooled library were used for homology searches and analyses with (1) 2,890 mapped 3' ESTs and corresponding 5' ESTs [6], (2) 5,006 FLcDNA sequences [36], (3) 502,895 barley EST sequences from Genbank, (4) Unigene #35 sequences from HarvEST [3], RAP2 rice gene nucleotide sequences (rap2_nuc_rep [23]), (6) rice amino acid sequences (rap2_orf_aa [23]), (7) the Triticeae Repeat Sequence Database (TREP, [37]), (8) RepeatMasker (repeat, tRNA, rRNA [38]), (9) Genscan_Arabidopsis/Maize [39], (10) gene prediction by GlimmerHMM [40], (11) DNA/GC content [41], (12) 6-frame AA translation [41] and AUGUSTUS for *ab initio *gene annotation [42]. A preliminary genome annotation browser was developed under the framework of Gbrowse [30]. A query search system using unique sequence or map position on the cMAP browser [32] was implemented on the Gbrowse system.

### Sequence data

8,583 sequence data from this article have been submitted to the DDBJ/EMBL/GenBank Data Libraries. Accession numbers are [DDBJ:BACC01000001-BACC01008583]

## Abbreviations

BAC: bacterial artificial chromosome; EST: expressed sequence tag; SNP: single nucleotide polymorphism

## Authors' contributions

KS planned the experiments and wrote the paper, YM did sequencing, NY developed libraries and HY analyzed data. All authors read and approved the final manuscript.

## Supplementary Material

Additional file 1 **Information of pooled barley BAC clone libraries and resulting sequences**. Excel file Click here for file

Additional file 2 **Name, lengh and map position of barley ESTs on chromosome 3H used for BAC clone selection and their homologous contigs from BAC sequencing**. Excel file Click here for file

Additional file 3 **Barley regions showing homology with rice genes (rap2 nucleotide sequences in each locus)**. Existence of full rice gene sequence on barley contig is confirmed by the start and end positions of rice gene on the respective contig sequence. Excel file Click here for file

Additional file 4 **Rice genes (rap2 nucleotide sequences in each locus) showing blastn homology to barley conting sequences**. Excel file Click here for file

Additional file 5 ***Brachypodium *genes (Bd1.0CDS) showing blastn homology to barley conting sequences**. Excel file Click here for file

Additional file 6 **1,474 contigs showing significant blastn score with barley full length cDNAs**. Excel file Click here for file

Additional file 7 **EST sequences used for BAC clone selection**. Multi-FASTA file. Click here for file
